# Efficacy and safety of patisiran for familial amyloidotic polyneuropathy: a phase II multi-dose study

**DOI:** 10.1186/s13023-015-0326-6

**Published:** 2015-09-04

**Authors:** Ole B Suhr, Teresa Coelho, Juan Buades, Jean Pouget, Isabel Conceicao, John Berk, Hartmut Schmidt, Márcia Waddington-Cruz, Josep M. Campistol, Brian R. Bettencourt, Akshay Vaishnaw, Jared Gollob, David Adams

**Affiliations:** Department of Public Health and Clinical Medicine, Umeå University, 901 87 Umeå, Sweden; Hospital de Santo António, Centro Hospitalar do Porto, 4099-001 Porto, Portugal; Servicio de Medicina Interna, Hospital Son Llatzer, Carretera Manacor KM, 7198 Palma de Mallorca, Spain; Hôpital de La Timone, 264 rue Saint Pierre, 13005 Marseille, France; Centro Hospitalar Lisboa Norte-Hospital de Santa Maria, Piso 7, Av, Prof Eqas Moniz, Lisboa, 1649-028 Portugal; Boston University, 72 East Concord Street, K-503, Boston, MA 02118 USA; Universitätsklinikum Münster, Transplant Hepatology, Domagkstr. 3A, Munster, 48149 Germany; Hospital Universitário Clementino Fraga Filho, Federal University of Rio de Janeiro, Ilha do Fundao, Rio de Janeiro, CEP21941-913 Brazil; Hospital Clinic, University of Barcelona, IDIBAPS, Escalera 12 (5 Planta), C/ Villarroel, 170, Barcelona, 8036 Spain; Alnylam Pharmaceuticals, 300 Third Street, Cambridge, MA 02142 USA; National Reference Center for FAP (NNERF)/ APHP/ INSERM U 1191/ Hôpital de Bicêtre, 78, rue du General Leclerc, 94275 Le Kremlin-Bicêtre, France

**Keywords:** Patisiran, RNA interference, Transthyretin-mediated familial amyloidotic polyneuropathy, Polyneuropathy, Hereditary disease, Genetic mutation, Phase II, Clinical trial

## Abstract

**Background:**

Transthyretin-mediated amyloidosis is an inherited, progressively debilitating disease caused by mutations in the transthyretin gene. This study evaluated the safety, tolerability, pharmacokinetics, and pharmacodynamics of multiple doses of patisiran (ALN-TTR02), a small interfering RNA encapsulated within lipid nanoparticles, in patients with transthyretin-mediated familial amyloid polyneuropathy (FAP).

**Methods:**

In this phase II study, patients with FAP were administered 2 intravenous infusions of patisiran at one of the following doses: 0.01 (*n* = 4), 0.05 (*n* = 3), 0.15 (*n* = 3), or 0.3 (*n* = 7) mg/kg every 4 weeks (Q4W), or 0.3 mg/kg (*n* = 12) every 3 weeks (Q3W).

**Results:**

Of 29 patients in the intent-to-treat population, 26 completed the study. Administration of patisiran led to rapid, dose-dependent, and durable knockdown of transthyretin, with the maximum effect seen with patisiran 0.3 mg/kg; levels of mutant and wild-type transthyretin were reduced to a similar extent in Val30Met patients. A mean level of knockdown exceeding 85 % after the second dose, with maximum knockdown of 96 %, was observed for the Q3W dose. The most common treatment-related adverse event (AE) was mild-to-moderate infusion-related reactions in 10.3 % of patients. Four serious AEs (SAEs) were reported in 1 patient administered 0.3 mg/kg Q3W (urinary tract infection, sepsis, nausea, vomiting), and 1 patient administered 0.3 mg/kg Q4W had 1 SAE (extravasation-related cellulitis).

**Conclusions:**

Patisiran was generally well tolerated and resulted in significant dose-dependent knockdown of transthyretin protein in patients with FAP. Patisiran 0.3 mg/kg Q3W is currently in phase III development.

**Trial registration number:**

NCT01617967.

**Electronic supplementary material:**

The online version of this article (doi:10.1186/s13023-015-0326-6) contains supplementary material, which is available to authorized users.

## Background

Transthyretin (TTR) is a tetrameric protein produced primarily in the liver. Mutations in the TTR gene destabilize the tetramer, leading to misfolding of monomers and aggregation into TTR amyloid fibrils (ATTR). Tissue deposition results in systemic ATTR amyloidosis [1–3]. Over 100 reported TTR mutations exhibit a spectrum of disease symptoms. The most common mutations associated with familial amyloid polyneuropathy (FAP) and ATTR-associated cardiomyopathy, respectively, are Val30Met [4] and Val122Ile [5].

Treatment options for FAP focus on stabilizing or decreasing the amount of circulating amyloidogenic protein. Orthotopic liver transplantation reduces mutant TTR levels [6], with improved survival reported in patients with early-stage FAP, although deposition of wild-type TTR may continue [7–12]. Tafamidis and diflunisal stabilize circulating TTR tetramers, which can slow the rate of disease progression [4, 13–15]. However, symptoms continue to worsen on treatment in many patients [4, 13–15], highlighting the need for new, disease-modifying treatment options for FAP.

RNA interference is a cellular process that controls gene expression, in which small interfering RNAs (siRNAs) mediate the cleavage of specific messenger RNAs (mRNAs) [16, 17]. Lipid nanoparticles (LNPs) deliver siRNAs to hepatocytes, resulting in the robust and durable reduction in expression (so-called “knockdown”) of gene targets across multiple species [18–23]. Patisiran (ALN-TTR02) comprises a TTR mRNA-specific siRNA formulated in LNPs [20]. A phase I ascending-dose study in healthy volunteers demonstrated rapid, dose-dependent, and durable knockdown of serum TTR with patisiran [20]. The objective of this study was to evaluate the safety, tolerability, pharmacokinetics (PK), and pharmacodynamics (PD) of multiple ascending intravenous (IV) doses of patisiran in patients with FAP.

## Methods

### Patients

Eligible patients were adults (≥18 years) with biopsy-proven ATTR amyloidosis and mild-to-moderate neuropathy; Karnofsky performance status ≥ 60 %; body mass index 17–33 kg/m^2^; adequate liver and renal function (aspartate transaminase [AST] and alanine transaminase [ALT] ≤ 2.5 × the upper limit of normal [ULN], total bilirubin within normal limits, albumin > 3 g/dL, international normalized ratio ≤ 1.2, serum creatinine ≤ 1.5 ULN); and seronegativity for hepatitis B virus and hepatitis C virus. Patients were excluded if they had a liver transplant; had surgery planned during the study; were HIV-positive; had received an investigational drug other than tafamidis or diflunisal within 30 days; had a New York Heart Association heart failure classification > 2; were pregnant or nursing; had known or suspected systemic bacterial, viral, parasitic, or fungal infections; had unstable angina; had uncontrolled clinically significant cardiac arrhythmia; or had a prior severe reaction to a liposomal product or known hypersensitivity to oligonucleotides.

### Study design

This was a multicenter, international, open-label, multiple-dose escalation phase II study of patisiran in patients with FAP. Cohorts of 3 patients received 2 doses of patisiran, with each dose administered as an IV infusion. Cohorts 1–3 received 2 doses of patisiran 0.01, 0.05, and 0.15 mg/kg every 4 weeks (Q4W), respectively; cohorts 4 and 5 both received 2 doses of patisiran 0.3 mg/kg Q4W. All patients in cohorts 6–9 received 2 doses of patisiran 0.3 mg/kg administered every 3 weeks (Q3W). As lipid-based delivery systems have previously been associated with adverse immune events [24, 25], all patients received premedication prior to each patisiran infusion consisting of dexamethasone, paracetamol (acetaminophen), an H2 blocker (e.g. ranitidine or famotidine), and an H1 blocker (e.g. cetirizine, hydroxyzine, or fexofenadine) to reduce the risk of infusion-related reactions (further details on premedication regimen are provided in Additional file 1). Patisiran was administered IV at 3.3 mL/min over 60 min, or over 70 min using a micro-dosing regimen (1.1 mL/min for 15 min followed by 3.3 mL/min for the remainder of the dose). Cumulative safety and tolerability data on all patients was reviewed by the Safety Review Committee (SRC). This study provides Class III evidence on the efficacy and safety of patisiran in patients with FAP.

### Standard protocol approvals, registrations, and patient consents

The study procedures (Clinicaltrials.gov identifier: NCT01617967) were approved by the ethics committee on human experimentation at each site. All patients provided written informed consent.

### Outcome measures

The primary study objective was to evaluate the safety and tolerability of multiple ascending doses of patisiran. Secondary objectives were to characterize the plasma and urine PK of patisiran, and to assess preliminary evidence of the PD effect of patisiran on serum total TTR protein levels.

Serum levels of total TTR protein were assessed for all patients using an enzyme-linked immunosorbent assay (ELISA). Additionally, wild-type and mutant TTR protein were separately and specifically measured in serum for patients with the Val30Met mutation using a proprietary mass spectrometry method (Charles River Laboratories, Quebec, Canada). Serum samples were collected at screening, and on Days 0, 1, 2, 7, 10, 14, 21, 22, 23 (Q3W only); 28, 29 (Q4W only); 30 (Q4W only); 31 (Q3W only); 35, 38 (Q4W only); and 42, 49, 56, 112, and 208 of follow-up.

Plasma concentration–time profiles were created for TTR siRNA, based on blood samples collected on Day 0 and at the following time points: pre-dose (within 1 h of planned dosing start), at end of infusion (EOI), at 5, 10, and 30 min, and at 1, 2, 4, 6, 24, 48, 168, 336, 504 (Day 21, Q3W regimen only), and 672 h (Day 28, Q4W regimen only) post-infusion. Additional samples were collected on Days 84 and 180 for the Q4W regimens, and on Days 35, 91, and 187 for the Q3W regimen. For cohorts 3–9, blood samples on Day 0 at EOI and 2 h post-infusion were also analyzed for both free and encapsulated TTR siRNA. Serum TTR siRNA was analyzed using a validated ATTO-Probe high-performance liquid chromatography (HPLC) assay (Tandem Laboratories, Salt Lake City, Utah, USA). PK analyses were conducted using non-compartmental and/or compartmental evaluation of TTR siRNA plasma concentration–time data to determine PK parameter estimates using the validated software program WinNonlin®. Urine samples were analyzed for levels of excreted TTR siRNA, and renal clearance was measured after dosing.

Serum levels of vitamin A and retinol binding protein (RBP) were measured by HPLC and nephelometry, respectively, at the same time points specified for total TTR (Biomins Specialized Medical Pathology, Lyon, France).

Safety evaluations included assessment of adverse events (AEs), electrocardiograms (ECGs), arterial oxygen saturation using pulse oximetry, vital signs, clinical laboratory safety tests, and physical examinations. AEs were defined as mild (easily tolerated with no disruption of normal daily activity), moderate (sufficient discomfort to interfere with daily activity), or severe (those which incapacitated and prevented usual activity). The numbers and percentages of patients with any treatment-emergent AE (TEAE), with any serious TEAE, with any TEAE leading to discontinuation of study medication, or with any TEAE considered a dose-limiting toxicity were summarized by dose cohort and overall. Dose-limiting toxicities included any of the following: any life-threatening toxicity; ALT and AST ≥ 5 × ULN or total bilirubin > 2.0 mg/dL; an infusion reaction that required hospitalization; and any other toxicity that in the opinion of the SRC precluded administration of a second dose.

### Statistical analyses

Based on the planned dose escalation scheme, we expected to enroll 27 patients. Patient populations included intent-to-treat (ITT, all patients who were enrolled and received study treatment) and per-protocol (PP, ITT patients with no major protocol violations). Safety measures were assessed in the ITT population. No substitutions were made to allow for missing data points.

Means and variances for TTR knockdown from baseline were calculated for the PP population, with baseline defined as the average of all pre-dose values. We used analysis of variance (ANOVA) and analysis of covariance (ANCOVA) to analyze the PD data (natural log transformed TTR relative to baseline), with Tukey’s post hoc tests of individual pairwise comparisons (between dose levels). Nadir TTR levels were defined as the minimum level per patient during the 28-day period (21-day period for Q3W group) after each dose administration (first-dose, second-dose periods: Days 1–28, 29–56, and Days 1–21, 22–42 for Q4W and Q3W groups, respectively). Relationships between TTR and RBP or vitamin A, relative to baseline, and the relationship between wild-type and V30M TTR levels, were explored via linear regression. We formally evaluated the dose proportionality of the patisiran component in PK parameters using a power model analysis. AEs were coded using the Medical Dictionary for Regulatory Activities coding system, version 15.0, and descriptive statistics provided for AEs, laboratory data, vital signs data, and ECG interval data. All statistical analyses were performed using SAS (version 9.3 or higher) and/or R (version 2.6 or higher).

## Results

### Baseline demographics and disease characteristics

A total of 29 patients were enrolled across 7 countries: Brazil, France, Germany, Portugal, Spain, Sweden, and the USA. All patients were white, 69 % were male, and the mean (standard deviation [SD]) age was 56 (15.6) years (Table 1). The majority of patients (76 %) had the Val30Met TTR mutation. A small proportion (14 %) of patients had walking difficulties requiring the use of a stick or crutch, with the remainder having unimpaired ambulation. The majority of patients were taking a concurrent TTR tetramer stabilizer, including 48 % on tafamidis and 24 % using diflunisal.

**Table 1:** Baseline demographics and disease characteristics (intent-to-treat population)

Parameter	0.01 mg/kg	0.05 mg/kg	0.15 mg/kg	0.3 mg/kg	0.3 mg/kg	All patients
	Q4W	Q4W	Q4W	Q4W	Q3W	(*n* = 29)
	(*n* = 4)	(*n* = 3)	(*n* = 3)	(*n* = 7)	(*n* = 12)	
Sex, n (%)						
Male	3 (75.0)	3 (100.0)	2 (66.7)	3 (42.9)	9 (75.0)	20 (69.0)
Female	1 (25.0)	0	1 (33.3)	4 (57.1)	3 (25.0)	9 (31.0)
Age, years						
Mean (SD)	65.8 (8.96)	55.7 (24.83)	41.7 (2.52)	58.7 (16.07)	53.8 (15.6)	55.6 (15.61)
Race, n (%)						
White/Caucasian	4 (100.0)	3 (100.0)	3 (100.0)	7 (100.0)	12 (100.0)	29 (100.0)
Country						
Portugal	0	1 (33.3)	3 (100.0)	2 (28.6)	3 (25.0)	9 (31.0)
Sweden	2 (50.0)	1 (33.3)	0	2 (28.6)	1 (8.33)	6 (20.7)
France	2 (50.0)	1 (33.3)	0	2 (28.6)	3 (25.0)	8 (27.6)
Brazil	0	0	0	0	1 (8.33)	1 (3.4)
Germany	0	0	0	0	1 (8.33)	1 (3.4)
Spain	0	0	0	0	3 (25.0)	3 (10.3)
United States	0	0	0	1 (14.3)	0	1 (3.4)
TTR genotype, n (%)						
Val30Met	2 (50.0)	2 (66.7)	3 (100.0)	6 (85.7)	9 (75.0)	22 (75.9)
Other^a^	2 (50.0)	1 (33.3)	0	1 (14.3)	3 (25.0)	7 (24.1)
Mean (SD) serum TTR at baseline, μg/mL	272.9 (98.96)	226.5 (12.67)	276.1 (7.65)	242.6 (38.30)	235.5 (44.45)	245.64 (49.44)
FAP stage^b^						
1	–	–	–	–	–	25 (86.2)
2	–	–	–	–	—	4 (13.8)
Prior exposure to ALN-TTR01^c^						
Yes	2 (50.0)	0	2 (66.7)	3 (42.9)	2 (16.67)	9 (31.0)
No	2 (50.0)	3 (100.0)	1 (33.3)	4 (57.1)	10 (83.33)	20 (69.0)
Concurrent TTR stabilizer use						
Diflunisal	2 (50.0)	1 (33.3)	0	3 (42.9)	1 (33.3)	7 (24.1)
Tafamidis	0	1 (33.3)	2 (66.7)	4 (57.1)	7 (66.7)	14 (48.3)

^a^Non-Val30Met mutations: Arg45Thr (*n* = 1), Phe46Leu (*n* = 1), Ser77Tyr (*n* = 2), Ser77Phe (*n* = 2), Tyr116Ser (*n* = 1)

^b^FAP stage: 1 = unimpaired ambulation, mostly mild neuropathy in lower limbs; 2 = assistance with ambulation required, mostly moderate neuropathy with progression to lower limbs, upper limbs, and trunk; 3 = wheelchair-bound or bedridden, severe neuropathy of all limbs

^c^ALN-TTR01 was the first-generation siRNA-LNP used in phase I studies in patients with ATTR

*ATTR* TTR amyloid fibrils, *FAP* familial amyloid polyneuropathy; *Q3W* every 3 weeks; *Q4W* every 4 weeks; *SD* standard deviation; *siRNA-LNP* small interfering RNA-lipid nanoparticles; *TTR* transthyretin

### Patient disposition

Of the 29 patients enrolled, all received study treatment and were included in the ITT population, and 26 patients completed the study. Two patients discontinued from the study after receiving only 1 dose of patisiran: 1 patient in the 0.01 mg/kg dosing group (cohort 1) discontinued following a protocol amendment (Additional file 1), and 1 patient in the 0.3 mg/kg Q3W group withdrew from the study because of an AE. Following the protocol-related patient discontinuation in the 0.01 mg/kg Q4W group, an additional patient was enrolled and included in this dose cohort. In addition to the 2 patients who discontinued after the first dose of patisiran, 1 patient in the 0.3 mg/kg Q4W group did not complete the study due to a protocol violation (missed follow-up assessment). This patient was excluded from PD analyses after Day 28 owing to an AE (extravasation of the second dose of patisiran), and an additional patient was recruited to the study.

Seven patients received patisiran 0.3 mg/kg Q4W (cohorts 4 and 5) and 4 patients received patisiran 0.01 mg/kg; all other study cohorts included 3 patients as described. Of the cohorts treated with patisiran 0.3 mg/kg, 10 patients received their infusions over 60 min, and 9 patients (all in the Q3W group) received the 70-min micro-dosing regimen. One patient in the 0.05 mg/kg Q4W cohort 2 did not meet the eligibility criteria because of liver function data, but was given a waiver to enter the study.

### Efficacy and PD

Mean (SD) baseline serum TTR protein levels were similar across the dose cohorts: 272.9 (98.86), 226.5 (12.67), 276.1 (7.65), 242.6 (38.30), and 235.5 (44.45) μg/mL for the 0.01, 0.05, 0.15, 0.3 Q4W, and 0.3 mg/kg Q3W dosage groups, respectively.

**Fig. 1:**
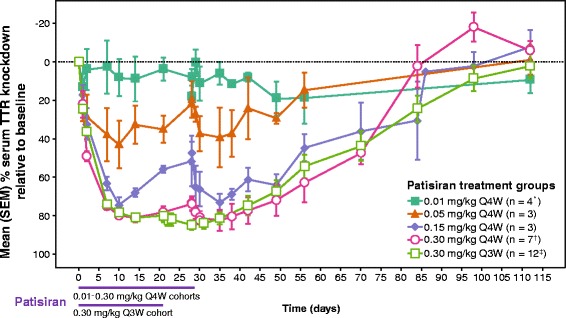
Dose response and duration of TTR knockdown. Mean (±SEM) percentage of baseline serum concentration–time profile. Q3W: every 3 weeks; Q4W: every 4 weeks; SEM: standard error of the mean; TTR: transthyretin. *Includes first dose data from additional patient prior to protocol amendment. ^†^Excludes post-Day 28 data from patient who experienced drug extravasation during second infusion. ^‡^One patient discontinued before the second dose of patisiran

**Fig. 2:**
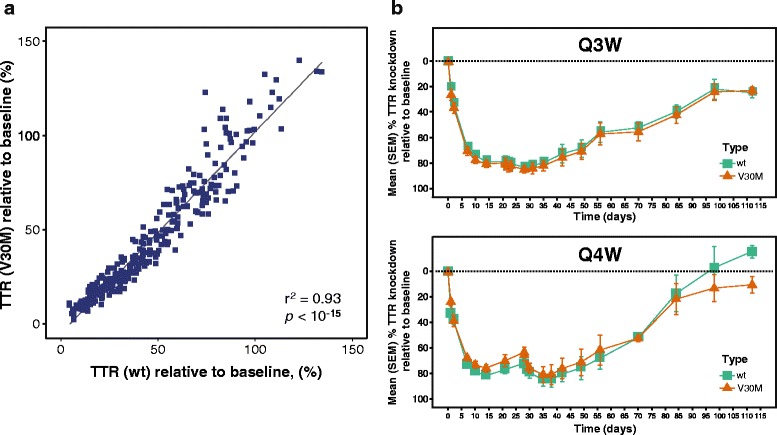
Effect of patisiran on wild-type and mutant TTR in patients with the Val30Met mutation. **a** All post-dose data. **b** Patisiran 300 mg/kg groups (error bars represent SEM). Q3W: every 3 weeks; Q4W: every 4 weeks; SEM: standard error of the mean; TTR: transthyretin; wt: wild-type

In comparison to the 0.01 mg/kg dose cohort, a significant reduction in nadir TTR levels (*p <* 0.001 by post hoc tests after ANCOVA) was observed after the first and second doses of patisiran in the 0.3 mg/kg Q4W and Q3W cohorts (Fig. 1 and Table 2). In patients given 0.3 mg/kg Q3W, mean ± SD TTR knockdown from baseline at nadir was 83.8 ± 5.1 % and 86.7 ± 7.0 % after the first and second doses, respectively, with maximum knockdown of 96 %. In patients given the same dose Q4W, mean ± SD TTR knockdown from baseline at nadir was 82.9 ± 5.4 % and 85.7 ± 9.6 % after the first and second doses, respectively, with maximum knockdown of 90.8 %. Mean TTR knockdown from baseline of ≥ 80 % was maintained between doses in the Q3W cohort, yet TTR levels recovered to < 80 % between doses with the Q4W regimen. In patients with the Val30Met mutation, a very similar degree of knockdown was observed for wild-type and mutant TTR (Fig. 2a and b). The levels of mutant and wild-type TTR knockdown were not assessed for each of the rarer non-Val30Met genotypes, although overall knockdown, as measured by ELISA, was indistinguishable between patients with Val30Met or non-Val30Met mutations. The level of serum TTR knockdown was highly correlated with the reduction in circulating level of RBP (r^2^ = 0.89, *p <* 10^−15^) and vitamin A (r^2^ = 0.89, *p <* 10^-15^) (Additional file 2: Figure S1).

**Table 2:** Serum TTR knockdown by dose group

Dose group (mg/kg)	Dose 1		Dose 2	
	Maximum TTR KD (%)	TTR KD at Nadir (Mean % ± SD)	Maximum TTR KD (%)	TTR KD at Nadir (Mean % ± SD)
0.01 Q4W (*n* = 4^a^)	37.8	22.1 ± 12.5	34.4	32.9 ± 2.3
0.05 Q4W (*n* = 3)	58.0	48.4 ± 16.2	58.5	46.9 ± 15.0
0.15 Q4W (*n* = 3)	81.7	74.5 ± 6.8***	86.0	77.0 ± 7.8
0.3 Q4W (*n* = 7^b^)	87.5	82.9 ± 5.4***	90.8	85.7 ± 9.6***
0.3 Q3W (*n* = 12^c^)	94.2	83.8 ± 5.1***	96.0	86.7 ± 7.0***

p values from ANCOVA models including baseline TTR as covariate and dose groups as factor; models significant at *p* < 0.001 for Dose 1, *p* < 0.001 for Dose 2

***p < 0.001 vs. 0.01 mg/kg group

^a^Includes first-dose data from additional patient prior to protocol amendment

^b^Excludes post-day 28 data from patient who experienced drug extravasation during second infusion

^c^One patient discontinued the study before second dose of patisiran

*ANCOVA* analysis of variance; *KD* knockdown; *Q3W* every 3 weeks; *Q4W* every 4 weeks; *SD* standard deviation; *TTR* transthyretin

Although patients taking tafamidis or diflunisal had significantly increased baseline levels of serum TTR compared with patients not taking stabilizer therapy (*p <* 0.001 by ANOVA) (Additional file 3: Figure S2a), patisiran administration resulted in a similar degree of TTR knockdown in these 2 patient groups (Additional file 3: Figure S2b).

### PK

Mean concentrations of the patisiran TTR siRNA component decreased after EOI (Additional file 4: Figure S3), and there was no accumulation of siRNA following the second dose on Day 21/28. Measurements of encapsulated versus un-encapsulated concentrations of TTR siRNA after each dose indicated stability of the circulating LNP formulation. For both the first and second doses, the mean values for maximum plasma concentration (C_max_) and area under the plasma concentration–time curve from zero to the last measurable time point (AUC_0–last_) increased in a dose-proportional manner over the dose range tested. C_max_ and AUC_0–last_ after doses 1 and 2 were comparable, with no accumulation. The median terminal half-life of patisiran at Day 0 and Days 21/28 was 39–59 h at doses > 0.01 mg/kg, and was relatively unchanged when comparing doses 1 and 2 for each dose cohort.

### Safety and tolerability

The most common TEAE related to study drug was mild-to-moderate infusion-related reaction (IRR), which occurred in 3/29 patients overall (10.3 %), all in the 0.3 mg/kg Q4W group (Table 3); none of these TEAEs led to discontinuation of treatment. IRR-associated symptoms were tachycardia, decreased oxygen saturation, dizziness, abdominal pain, bronchospasm, dyspnea, erythema, chills, pallor, pyrexia, and tachypnea. For 1 patient with a mild reaction, the event was managed by prolonging the IV infusion time. Of note, no IRRs were reported in the patient cohort that received 0.3 mg/kg Q3W (n = 12), 9 of whom received their infusion with the micro-dosing regimen administered over 70 min. Aside from IRRs, no drug-related TEAE was observed in more than 1 patient per dosage group across the study.

**Table 3:** Treatment-emergent adverse events related or possibly related to patisiran (ITT [safety] population)

Preferred term, n (%)	Patisiran					
	0.01 mg/kg	0.05 mg/kg	0.15 mg/kg	0.3 mg/kg	0.3 mg/kg	
	Q4W	Q4W	Q4W	Q4W	Q3W	Overall
	(*n* = 4)	(*n* = 3)	(*n* = 3)	(*n* = 7)	(*n* = 12)	(*n* = 29)
Infusion-related reaction	0	0	0	3 (42.9)	0	3 (10.3)
Back pain	0	0	0	2 (28.6)	0	2 (6.9)
Asthenia	0	0	0	1 (14.3)	1 (8.3)	2 (6.9)
Leukocytosis	0	0	0	0	1 (8.3)	1 (3.4)
Neutrophilia	0	0	0	0	1 (8.3)	1 (3.4)
Cellulitis^a^	0	0	0	1 (14.3)	0	1 (3.4)
Lymphangitis	0	0	0	1 (14.3)	0	1 (3.4)
Polyuria	0	0	0	1 (14.3)	0	1 (3.4)
Nausea/vomiting	0	0	0	0	1 (8.3)	1 (3.4)
Facial erythema	0	0	0	0	1 (8.3)	1 (3.4)
Dry mouth	0	0	0	0	1 (8.3)	1 (3.4)
Pyrexia	0	0	0	0	1 (8.3)	1 (3.4)
Dysphagia	0	0	0	0	1 (8.3)	1 (3.4)

^a^Due to drug extravasation

*ITT* intent-to-treat; *Q3W* every 3 weeks; *Q4W* every 4 weeks

There were no dose-limiting toxicities or deaths due to TEAEs reported during the course of the study. The majority of TEAEs were of mild or moderate intensity. Four serious AEs (SAEs) were reported in 1 patient in the 0.3 mg/kg Q3W group (urinary tract infection, sepsis, nausea, and vomiting), and the patient withdrew from the study because of the nausea and vomiting. An additional SAE (extravasation-related cellulitis) was recorded in 1 patient in the 0.3 mg/kg Q4W group. The SAEs of nausea and vomiting, and cellulitis were each recorded by the Investigator as related to the study drug.

No clinically significant changes in liver function tests, renal function, or hematologic parameters were recorded. Transient increases in white blood cell count were observed approximately 24 h after each patisiran infusion, which were considered related to dexamethasone premedication. There were no substantial changes in serum G-CSF, IFN-α, IFN-γ, IL-1b, IL-12, and TNF-α cytokines. Values were below the lower limit of detection for most patients at the majority of time points. Transient increases in mean levels of IL-6, IL-1RA, and IP10 were observed after patisiran infusion in the 0.3 mg/kg Q4W group (and in the 0.3 mg/kg Q3W group for IL-6), although levels returned to baseline within 24 h. Transient increases in mean levels of complement factor Bb were also seen after infusion of patisiran doses 0.05–0.3 mg/kg. None of these elevations were associated with AEs.

## Discussion

These phase II data demonstrate that administration of patisiran to patients with FAP led to robust, dose-dependent, and statistically significant knockdown of serum TTR protein levels. Mean sustained reduction in TTR of >80 % was achieved with 2 consecutive doses of patisiran 0.3 mg/kg dosed every 3–4 weeks, with a maximum knockdown of 96 % achieved in the Q3W group. These knockdown rates are consistent with the rates observed in the single ascending dose, placebo-controlled phase I study of patisiran [20]. Evidence from other systemic amyloidotic diseases indicates that as little as 50 % reduction of the disease-causing protein can result in clinical disease improvement or stabilization [26, 27], illustrating the potential of this therapy. The degree of TTR knockdown with patisiran was not affected by patients taking tafamidis or diflunisal, suggesting that these TTR stabilizer drugs do not interfere with the pharmacologic activity of patisiran. In patients with the Val30Met mutation, patisiran suppressed production of both mutated and wild-type TTR; the latter remains amyloidogenic in patients with late-onset FAP after liver transplantation [28, 29].

Multiple doses of patisiran were shown to be generally safe and well tolerated in this study. The majority of AEs were mild or moderate in severity and no dose-limiting toxicities were observed. While IRRs were the most common drug-related TEAE seen at 0.3 mg/kg, no IRRs were reported in the 0.3 mg/kg group that received the micro-dosing infusion regimen over 70 min. Premedication was used to reduce the risk of IRRs, and is thus considered in assessment of the tolerability of this regimen. Glucocorticoids such as dexamethasone can increase white blood cell levels [30], as observed transiently in this study, and TEAEs potentially related to premedication will be monitored during longer term patisiran administration. The generally favorable tolerability profile observed in this study, with most patients receiving TTR stabilizers, is encouraging with respect to the potential concurrent use of these agents with patisiran.

This multi-dose study defined patisiran 0.3 mg/kg Q3W delivered using the 70-min micro-dosing regimen as the formulation and dosage to administer in the ongoing open-label extension (OLE) phase of patients with FAP. Whilst both Q3W and Q4W achieved potent TTR knockdown, the level of TTR suppression was better maintained between doses with the Q3W schedule, supporting its future investigation. Preliminary data from the OLE study show sustained TTR knockdown of ~80 % after 168 days (9 doses), with no reported SAEs (n = 27) [31]. The OLE study preliminary safety data are consistent with the tolerability of patisiran observed in this trial, with TEAEs all mild or moderate in severity [31].

Patient baseline demographics and disease characteristics in the present study were similar to those reported for other recent FAP studies. In particular, the mean age (55.6 years) was comparable to that reported for a large natural history study of patients with FAP (56.4 years; n = 283) [32] and for a phase II/III diflunisal study (59.7 years; *n* = 130) [15], but was greater than the 39.8/38.4 years reported for the tafamidis/placebo arms in the tafamidis phase II/III study (*n* = 125) [14]. Some 75.9 % of patients in the present study had the Val30Met mutation compared with 58.5 % (161/275 evaluable patients), 54.6 %, and 100 % in the natural history, diflunisal, and tafamidis studies, respectively [14, 15, 32].

The results of this study supported the initiation of a phase III study of patisiran. APOLLO is a randomized, placebo-controlled, phase III study of patisiran 0.3 mg/kg Q3W administered using the 70-min micro-dosing schedule in recruiting patients with Val30Met or non-Val30Met FAP who are not taking TTR stabilizers. The primary study endpoint is disease progression, measured as the change from baseline in mNIS + 7 score at 18 months (Clinical trial identifier NCT01960348). Secondary endpoints include measures of quality of life and disease burden, changes from baseline in motor and autonomic neuropathy measures, and safety.

## Conclusion

In conclusion, the results of this phase II study provide evidence that the investigational agent patisiran was generally well tolerated and effective in reducing both mutant and wild-type TTR levels in patients with FAP. The 0.3 mg/kg Q3W dosing schedule is under investigation in the phase III APOLLO trial.

## Additional files

Additional file 1:
**Supplemental materials.** (DOCX 36 kb) Additional file 2: Figure S1. Correlation of serum TTR knockdown with change from baseline in serum vitamin A and RBP. RBP: retinol binding protein; TTR: transthyretin. (DOCX 235 kb) Additional file 3: Figure S2. TTR knockdown with patisiran in patients taking a tetramer stabilizer. (a) Baseline TTR levels by stabilizer use (all cohorts). (b) TTR knockdown by patisiran (0.3 mg/kg cohorts; error bars represent SEM). SEM: standard error of the mean; TTR: transthyretin. (DOCX 260 kb) Additional file 4: Figure S3. Mean (±SD) plasma concentration–time profiles of patisiran following 1 h intravenous infusion (semi-log scale) for: (a) First (Day 0) and (b) second (Day 21/28) doses. Q3W: every 3 weeks; Q4W: every 4 weeks; SD: standard deviation. (DOCX 196 kb)

## Abbreviations

AE: Adverse event
ALT: Alanine transaminase
ANCOVA: Analysis of covariance
ANOVA: Analysis of variance
AST: Aspartate transaminase
ATTR: TTR amyloid fibrils
AUC_0-last_: Area under the plasma concentration–time curve from zero to the last measurable time point
C_max_: Maximum plasma concentration
ECG: Electrocardiograms
EOI: End of infusion
FAP: Familial amyloid polyneuropathy
HPLC: High-performance liquid chromatography
IRR: Infusion-related reaction
ITT: Intent-to-treat
IV: Intravenous
KD: Knockdown
LNP: Lipid nanoparticles
OLE: Open-label extension
PD: Pharmacodynamics
PK: Pharmacokinetics
PP: Per-protocol
Q3W: Every 3 weeks
Q4W: Every 4 weeks
RBP: Retinol binding protein
SAE: Serious AE
SD: Standard deviation
siRNA: Small interfering RNA
siRNA-LNP: Small interfering RNA-lipid nanoparticle
SRC: Safety Review Committee
TEAE: Treatment-emergent AE
TTR: Transthyretin
ULN: Upper limit of normal
