# Supplementary material for: Efficacy and safety of patisiran for familial amyloidotic polyneuropathy: a phase II multi-dose study
Source: Orphanet J Rare Dis. 2015 Sep 4;10:109. doi: 10.1186/s13023-015-0326-6 (PMC4559363; doi:10.1186/s13023-015-0326-6)
Supplement: Additional file 1: — Supplemental materials. (DOCX 36 kb) [file 13023_2015_326_MOESM1_ESM.docx]

SUPPLEMENTAL MATERIALS

Protocol amendments

Two main protocol amendments were made, and a further 2 minor amendments (1.1 and 2.1) were implemented in France only. The main additions in Amendment 1 were based on new non-clinical toxicology data from the non-human primate study and favorable clinical data from the phase I trials ALN-TTR02 and ALN-PCS02. In summary, Amendment 1 removed the requirement for the SRC to review safety data on all patients within a dose level between the first and second doses of study drug; removed the 0.5 mg/kg dose cohort; extended the post-dose on-site observation period from 6 h to 24 h post-infusion; added additional safety evaluations; and added specific instructions for dosing of patients who weighed ≥ 105 kg. Amendment 2 added the every 3 weeks dosing regimen, and the slower infusion rate (70-minute micro-dosing regimen).

SUPPLEMENTAL MATERIALS

Premedication regimen

Patients received the following premedication regimen: dexamethasone (oral: 8 mg the evening before dosing and 20 mg 30–60 minutes before starting the patisiran infusion; or intravenous: 10 mg ≥ 60 minutes before the patisiran infusion), paracetamol (oral: 500 mg the evening before dosing and 30–60 minutes before the patisiran infusion; or 500 mg ≥60 min before the patisiran infusion), H2 blocker (oral: ranitidine 150 mg, famotidine 20 mg, or equivalent, the evening before dosing and 30–60 minutes before starting the patisiran infusion; or intravenous: ranitidine 50 mg, famotidine 20 mg, or equivalent, ≥ 60 minutes before the patisiran infusion), H1 blocker (oral: cetirizine 10 mg or hydroxyzine/fexofenadine 25 mg, the evening before dosing and 30–60 minutes before the patisiran infusion; or diphenhydramine 50 mg or equivalent, ≥60 minutes before the patisiran infusion).
