# Supplementary material for: Efficacy and safety of patisiran for familial amyloidotic polyneuropathy: a phase II multi-dose study
Source: Orphanet J Rare Dis. 2015 Sep 4;10:109. doi: 10.1186/s13023-015-0326-6 (PMC4559363; doi:10.1186/s13023-015-0326-6)

**Figure S1 Correlation of serum TTR knockdown with change from baseline in serum vitamin A and RBP.**

RBP: retinol binding protein; TTR: transthyretin.


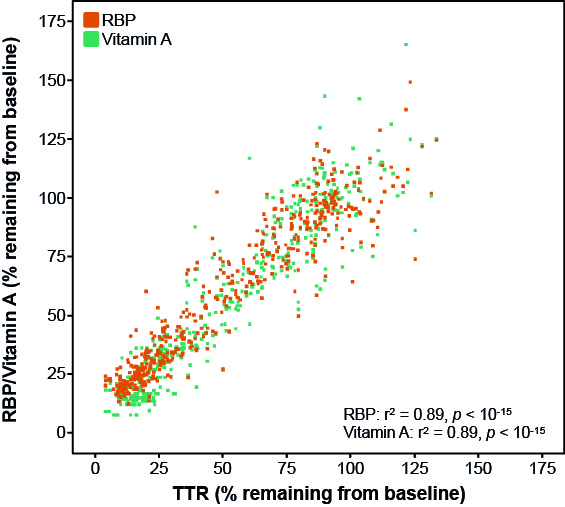

Supplement: Additional file 2: Figure S1. — Correlation of serum TTR knockdown with change from baseline in serum vitamin A and RBP. RBP: retinol binding protein; TTR: transthyretin. (DOCX 235 kb) [file 13023_2015_326_MOESM2_ESM.docx]
