# Supplementary material for: Efficacy and safety of patisiran for familial amyloidotic polyneuropathy: a phase II multi-dose study
Source: Orphanet J Rare Dis. 2015 Sep 4;10:109. doi: 10.1186/s13023-015-0326-6 (PMC4559363; doi:10.1186/s13023-015-0326-6)

**Figure S2 TTR knockdown with patisiran in patients taking a tetramer stabilizer.**

(a) Baseline TTR levels by stabilizer use (all cohorts). (b) TTR knockdown by patisiran (0.3 mg/kg cohorts; error bars represent SEM).

SEM: standard error of the mean; TTR: transthyretin.


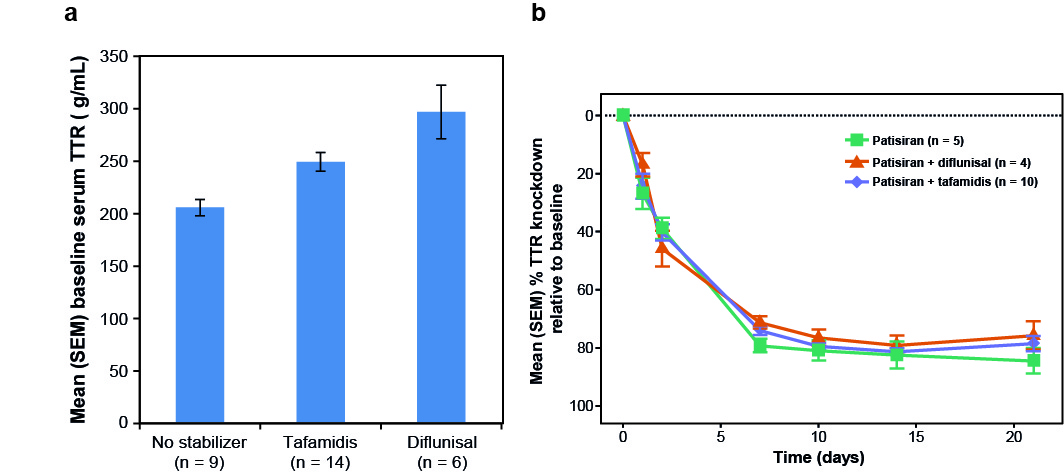

Supplement: Additional file 3: Figure S2. — TTR knockdown with patisiran in patients taking a tetramer stabilizer. (a) Baseline TTR levels by stabilizer use (all cohorts). (b) TTR knockdown by patisiran (0.3 mg/kg cohorts; error bars represent SEM). SEM: standard error of the mean; TTR: transthyretin. (DOCX 260 kb) [file 13023_2015_326_MOESM3_ESM.docx]
