# Supplementary material for: Efficacy and safety of patisiran for familial amyloidotic polyneuropathy: a phase II multi-dose study
Source: Orphanet J Rare Dis. 2015 Sep 4;10:109. doi: 10.1186/s13023-015-0326-6 (PMC4559363; doi:10.1186/s13023-015-0326-6)

**Figure S3 Mean (±SD) plasma concentration–time profiles of patisiran following 1 h intravenous infusion (semi-log scale) for:**

(a) First (Day 0) and (b) second (Day 21/28) doses.

Q3W: every 3 weeks; Q4W: every 4 weeks; SD: standard deviation.


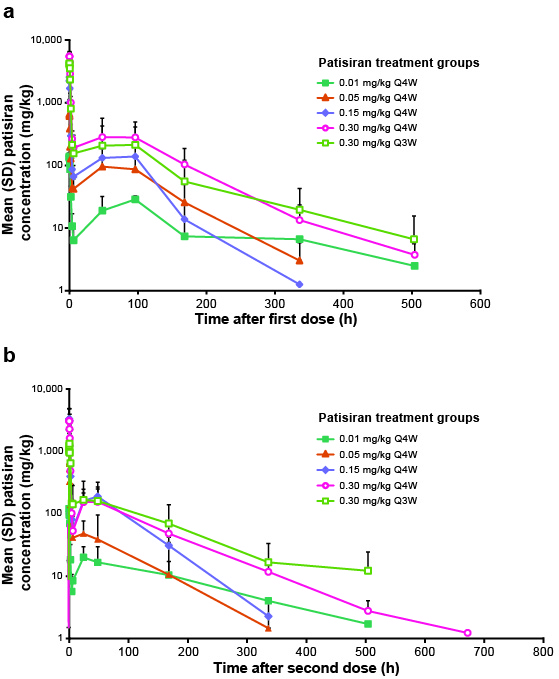

Supplement: Additional file 4: Figure S3. — Mean (±SD) plasma concentration–time profiles of patisiran following 1 h intravenous infusion (semi-log scale) for: (a) First (Day 0) and (b) second (Day 21/28) doses. Q3W: every 3 weeks; Q4W: every 4 weeks; SD: standard deviation. (DOCX 196 kb) [file 13023_2015_326_MOESM4_ESM.docx]
